# Neutrophil to lymphocyte count ratio performs better than procalcitonin as a biomarker for bacteremia and severe sepsis in the emergency department

**DOI:** 10.1186/cc14146

**Published:** 2015-03-16

**Authors:** LL Ljungström, D Karlsson, A Pernestig, R Andersson, G Jacobsson

**Affiliations:** 1 Skaraborg Hospital Skövde, Sweden; 2 School of Biosciences, University of Skövde, Sweden; 3 Institute of Biosciences, Sahlgrenska Academy at the University of Gothenburg, Sweden

## Introduction

The objective of this study was to evaluate the neutrophil to lymphocyte count ratio (NLCR) versus procalcitonin (PCT) in diagnosing bacteremia in the emergency department (ED). The NLCR is a biomarker that appears early in the course of the acute inflammatory response and reaches maximum levels within 4 hours after onset. An elevated NLCR has been shown to correlate to bacteremia at a cutoff level of >10 [1]. It is rapidly analyzed on a full blood cell count at low cost. The lowest recommended cutoff level for PCT is <0.5 ng/ml.

## Methods

We randomly chose 425 patients from a 9-month epidemiologic study on the incidence of community-onset severe sepsis and septic shock in western Sweden 2011 to 2012. In total, 207 had severe sepsis and 218 had sepsis, mean age 71.2 versus 64.2 years; males 51%. Sampling was made on arrival in the ED. The NLCR was analyzed immediately, PCT later on plasma frozen at -80°C. A total of 122/425 patients had bacteremia, 72 (35%) in the severe sepsis group versus 50 (23%) in the sepsis group. Most common findings were *Escherichia coli *(*n *= 33), *Staphylococcus aureus *(*n *= 24), streptococcal spp. (*n *= 33) and other enterobacteriacae spp. (*n *= 17).

## Results

The NLCR shows significantly higher sensitivity than PCT at recommended cutoff levels for bacteremia. Interestingly, this is true even for all 207 patients with severe sepsis, irrespective of bacteremia or not. Sensitivity figures with 95% confidence interval: bacteremia (*n *= 122): NLCR 80% (0.73 to 0.87) versus PCT 66% (0.58 to 0.75), *P *= 0.01; severe sepsis with bacteremia (*n *= 72): NLCR 85% (0.77 to 0.93) versus PCT 70% (0.59 to 0.81), *P *= 0.03; and severe sepsis but no bacteremia (*n *= 135): NLCR 71% (0.65 to 0.77) versus PCT 61% (0.54 to 0.68), *P *= 0.03.

## Conclusion

The NLCR can be used in the ED as a biomarker for bacteremia as well as severe sepsis and seems to perform as well as or even better than PCT in this setting. Rapid response, low cost and no need for extra sampling make it useful as a screening tool.
